# Does Physical Activity Affect Clinical Symptoms and the Quality of Life of Mild-Infected Individuals with COVID-19 in China? A Cross-Sectional Study

**DOI:** 10.3390/healthcare11152163

**Published:** 2023-07-30

**Authors:** Rong Wang, Yuanyuan Jia, Tingting Sun, Bing Ruan, Huixuan Zhou, Laikang Yu, Xiao Hou

**Affiliations:** 1 School of Sport Science, Beijing Sport University, Beijing 100084, China; 2020011945@bsu.edu.cn (R.W.);; 2 Key Laboratory of Sports and Physical Health Ministry of Education, Beijing Sport University, Beijing 100084, China; 3 School of Sport Medicine and Rehabilitation, Beijing Sport University, Beijing 100084, China; 4 Department of Sports Performance, Beijing Sport University, Beijing 100084, China

**Keywords:** physical activity, exercise, COVID-19, SARS-CoV-2, quality of life, clinical symptom

## Abstract

Background: Few studies have identified the links between physical activity (PA), clinical symptoms, and the quality of life (QoL) among mildly infected individuals with COVID-19. This cross-sectional study aims to evaluate how PA levels before infections affect the infectious symptoms and the QoL in mildly infected patients with COVID-19. Methods: An online questionnaire link including participants’ sociodemographic and anthropometric characteristics, clinical symptoms during the COVID-19 infectious period, the QoL of the worst symptomatic day, and PA in the last seven days before COVID-19 infections was disclosed. Logistic regression and multiple linear regression analyses were applied to assess the relationships between PA levels in the last seven days before infections and COVID-19-related outcomes. The level of statistical significance was set at *p* < 0.05. Results: Compared to the low-PA-level group, the moderate-PA-level group presented a higher risk of headaches (OR = 1.34, 95% CI = 1.03 to 1.75, and *p* = 0.03) and the high-PA-level group presented a higher risk of muscle/body aches (OR = 1.42, 95% CI = 1.04 to 1.93, and *p* = 0.03). The adjusted linear regression analysis showed that no associations were found between PA levels in the last seven days before infections and the QoL index value on the worst symptomatic day (moderate-PA-level group: β = −0.04, and *p* = 0.08; high-PA-level group: β = −0.04, and *p* = 0.17). However, for the mobility and usual activities dimensions of EQ-5D-5L, the lower-PA-level group had a lower burden of QoL than the higher-PA-level group did on the worst-symptomatic day. Conclusions: Among mildly infected patients with COVID-19, a higher PA level is associated with a higher risk of experiencing clinical symptoms and a lower QoL.

## 1. Introduction

In December 2019, the first case of novel coronavirus was detected in China, with the virus spreading rapidly across various countries and regions around the world [1]. Subsequently, the World Health Organization (WHO) characterized the outbreak of the coronavirus disease 2019 (COVID-19) as a pandemic [2]. By 1 January 2023, more than 656 million confirmed cases and over 6.6 million deaths were documented worldwide [3]. Due to the COVID-19 pandemic, lots of countries implemented restriction policies (e.g., home isolation, school suspension, and bans of mass gatherings) to curb population flow and reduce the viral spread [4]. China, as a country with a massive and dense population, has also taken several measures to limit the risk of infection from COVID-19, such as self-quarantining from close contact and closing public places such as restaurants, shopping malls, and cinemas [5].

As the virus variants appeared and the virulence weakened, several countries ended the mass lockdowns. The Chinese government also optimized COVID-19 epidemic prevention measures to match the features of the omicron variant at the end of 2022. Although several sub-lineages of the virus attenuate virulence, leading to a significant reduction in severely affected patients with COVID-19, the relaxed restrictions have still caused numerous individuals to become inflicted with asymptomatic infections or mild symptoms of COVID-19. Chinese Center for Disease Control and Prevention (China CDC) has reported that the number of people who tested positive for COVID-19 through a polymerase chain reaction (PCR) shows an increasing trend since 9 December 2022, with the number of positive cases peaking on 22 December at 6.94 million [6].

With the relaxation of global lockdowns, mild infections of COVID-19 have become the norm and even superinfections also happen occasionally. In addition to taking precautions such as wearing a mask and keeping a safe distance, it is also essential to take proactive measures such as strengthening physical health to deal with the hazards posed by COVID-19. Physical activity (PA), as a non-pharmacological treatment, might be a good choice. It has been commonly indicated that PA or exercise after COVID-19 infections can accelerate the recovery of the sequelae induced by novel coronaviruses [7,8,9]. For example, continuous aerobic training combined with moderate-intensity resistance training in the healing phase has been proven helpful to reduce perceived fatigue and enhance the quality of life (QoL) of COVID-19-infected patients [7]. In addition, Gil et al. have also proposed that COVID-19 survivors with acute sequelae have greater odds of being physically inactive relative to those without any persistent symptoms (OR = 1.57, 95% CI = 1.04 to 2.39, and *p* = 0.032) [10]. Although it has been well-established that PA or exercise after COVID-19-infections can decrease the health-related damage caused by infections, few studies have reported whether PA before COVID-19 infections is beneficial to decreasing the number of symptoms and enhancing the QoL of COVID-19 patients during the infectious stage. Hence, it is necessary to explore how PA levels before COVID-19 infections affect patients’ clinical symptoms and the QoL during the infectious period.

In fact, there are individual studies evaluating the effect of PA before COVID-19 infections on several clinical outcomes [11,12,13,14]. However, they mainly focused on the relationships between PA levels and the prevalence of COVID-19-associated hospitalization in severely infected individuals or clinical outcomes (e.g., mortality) among hospitalized patients with moderate-to-severe COVID-19 symptoms [11,12]. For example, Pinto et al. recruited 209 hospitalized patients with moderate-to-severe COVID-19 symptoms from Brazil and suggested that no independent associations are found between PA and mortality, admission to the intensive care unit (ICU), or mechanical ventilation requirement [12]. Additionally, there is a growing body of research examining the impact of COVID-19 on clinical outcomes, particularly in athlete populations [15]. These studies have shed light on the adverse physiological effects experienced by infected individuals, which include reduced respiratory muscle strength, compromised pulmonary function, muscular and neurological damage, and negative psychological effects. Furthermore, the detrimental consequences of these physiological impairments have a significant impact on the overall QoL of those affected [15,16]. Nevertheless, as mentioned above, mild symptoms of COVID-19 are prevalent due to the reduced virulence. Thus, the links between PA before infections, clinical symptoms, and the QoL among mildly infected patients with COVID-19 should be explored. Additionally, lots of studies have confirmed that physical inactivity has been regarded as a predisposing factor to acquired infections [17]. Consequently, we aimed to examine whether or not the daily PA levels prior to infections were predictors of clinical symptoms and the QoL of infected people with mild COVID-19.

The objective of this study is to evaluate the associations between PA, clinical symptoms, and the QoL among mildly infected individuals with COVID-19 through a cross-sectional study. Understanding the influence of PA levels before COVID-19 infections on clinical symptoms and the QoL during the infectious period can help patients relieve pain and reduce the discomfort caused by symptoms. It can also assist public health authorities in implementing precautionary policies in response to the normalization of mild infections of COVID-19. We hypothesized that a higher PA level would be associated with lower risks of clinical symptoms and higher QoL scores.

## 2. Methods

### 2.1. Participants and Study Design

A cross-sectional design was used to gather data through an online questionnaire survey. Chinese individuals who had tested positive for COVID-19 via a PCR or antigen detection were invited to participate in this survey. Moreover, only the data of mild-infected individuals were analyzed. According to an adapted version of the National Institute of Health (NIH) symptom severity classification scheme, mildly infected patients with COVID-19 are regarded as those who have any of the various signs and symptoms of COVID-19 (e.g., fever, cough, sore throat, malaise, headaches, muscle pain, nausea, vomiting, diarrhea, or loss of taste and smell) but who do not have shortness of breath, dyspnea, or abnormal chest imaging [18].

To complete data collection, we disclosed an electronic link to the questionnaire through a social media platform, “WeChat”, from 5 January 2023 to 15 February 2023. The questionnaire included questions concerning participants’ sociodemographic and anthropometric characteristics, clinical symptoms during the COVID-19 infectious period, the QoL on the worst symptomatic day, and PA in the last seven days before COVID-19 infections. Missing values were avoided by setting the required fields for every question in the online survey.

The study protocol was approved by the Ethics Committee of Sport Science Experiment, Beijing Sport University (2022035H). Participants were informed that their participation was completely voluntary and that they could withdraw without providing any justification or not answer any survey questions at any time. Participants were assured of the confidentiality of their information. Informed consent was offered through the home page of the online survey and was collected when participants completed the questionnaire.

### 2.2. Sociodemographic and Anthropometric Characteristics

The self-reported data including age, gender, height, weight, educational levels, marital status, employment, history of chronic diseases, and vaccination status were collected. The body mass index (BMI) was calculated using the following formula: BMI = body weight/height^2^ (unit: kg/m^2^). The educational levels were classified as follows: “primary or below”, “junior high”, “senior high”, “college”, and “postgraduate or above”. The marital status categories were as follows: “single”, “married”, “divorced”, and “widowed”. The employments were categorized as follows: “employed”, “student”, “retired”, and “others”. The vaccination status was classified as follows: “unvaccinated”, “one dose of vaccine”, “two doses of vaccine”, “three doses of vaccine”, and “four doses of vaccine”.

### 2.3. Clinical Symptoms

The clinical symptoms that occurred during the COVID-19 infectious phase, including “sore throat”, “fever or chills”, “cough”, “headaches”, “self-perceived fatigue”, “anorexia”, “congestion or runny nose”, “muscle or body aches”, “loss of smell”, “loss of taste”, “nausea”, “diarrhea or vomiting”, and “conjunctivitis” were also investigated.

### 2.4. QoL

The QoL on the worst symptomatic day during the COVID-19 infectious period was measured using the five-level EuroQol five-dimensional questionnaire (EQ-5D-5L). This questionnaire consists of five dimensions (mobility, MO; self-care, SC; usual activities, UA; pain and discomfort, PD; anxiety and depression, AD), each of which has five severity levels. Thus, a five-digit number was used to show the functional levels for the dimensions in the order of presentation (MO, SC, UA, PD, and AD). The number “11111” means no problems on all of the five dimensions, whereas the number “55555” represents extreme problems on all of the five dimensions. Based on the EQ-5D-5L User Guide, the five-digit code could be represented by a single summary number (index value) [19]. Finally, we converted the five-digit code of EQ-5D-5L health states into an index value using the EQ-5D-5L value set for China [20]. Another part of the EQ-5D-5L is a visual analogue scale (EQ VAS), which is numbered from 0 (which means the worst health state you can imagine) to 100 (which means the best health state you can imagine). Participants were also required to score the perception of their overall health state on the worst symptomatic day while suffering from COVID-19.

### 2.5. PA

The International Physical Activity Questionnaire—Short Form (IPAQ-SF) was adopted to measure PA levels in the last seven days before COVID-19 infections. Its reliability and validity have been tested and verified in China [21,22]. The IPAQ-SF contains seven questions, which could be used to obtain different levels of PA, such as the weekly minutes of vigorous-intensity PA, moderate-intensity PA, walking, as well as sedentary behavior on a weekday.

According to participants’ recall, we retrospectively measured their pre-COVID-19 PA levels in the last seven days. Based on the guideline for data processing and the analysis of IPAQ [23], the total volume of activities could be converted into metabolic equivalents of task (MET) minutes. The formulae for the computation of MET minutes are shown in the following: (1) walking MET-minutes/week = 3.3 × walking minutes × walking days; (2) moderate MET-minutes/week = 4.0 × moderate-intensity activity minutes × moderate days; (3) vigorous MET-minutes/week = 8.0 × vigorous-intensity activity minutes × vigorous-intensity days.

According to the IPAQ guidelines, we classified the participants into three groups: the low-PA-level group, the moderate-PA-level group, and the high-PA-level group if they met Category 1, Category 2, and Category 3, respectively. The classification of PA levels based on IPAQ is shown in Table 1.

### 2.6. Statistical Analysis

The descriptive statistical analysis was conducted to analyze participants’ sociodemographic and anthropometric characteristics. Using the Kolmogorov–Smirnov test and histograms to assess the data’s normality. The continuous variables were reported as means with standard deviations (Mean ± SD) and the categorical variables were presented using frequencies (n) and percentages (%). Binary logistic regression was used to analyze the associations between PA levels in the last seven days before COVID-19 infections and clinical symptoms. The multiple linear regression analysis was used to test the links between PA levels in the last seven days before COVID-19 infections and the QoL index value. Multinomial logistic regression was used to assess the relationships between PA levels in the last seven days before COVID-19 infections and the five dimensions of the EQ-5D-5L. Regression models were conducted without adjustments (unadjusted models) and with adjustments for potential confounders (i.e., age, gender, BMI, history of chronic diseases, smoking status, and drinking status) (adjusted models). All the data were analyzed using Statistical Package of the Social Sciences (SPSS version 26.0; IBM Corp., Armonk, NY, USA). The significance level was set at *p* < 0.05.

## 3. Results

Table 2 shows the characteristics of the participants. Of the 1299 questionnaires returned, 1239 were valid (95.4%). Finally, a total of 1239 participants (age: 26.02 ± 10.27 years old; BMI: 22.39 ± 5.35 kg/m^2^) were included in our study. More than half of the participants were female (*n* = 756, 61.0%). For educational levels, only six participants had primary education or below (0.5%), 41 participants completed junior high (3.3%), 92 participants completed senior high (7.4%), 960 participants completed college (77.5%), and 140 participants were post-graduates or above (11.3%). The majority of participants were single individuals (*n* = 959, 77.4%) and students (*n* = 829, 66.9%). Among the 13 kinds of COVID-19 symptoms listed above, the majority of participants (*n* = 789, 63.7%) reported having less than seven symptoms, while 450 participants (36.3%) reported having at least seven symptoms. Additionally, only 19.0% of participants did exercise outdoors during the COVID-19 pandemic. For vaccination status, 21 participants were unvaccinated (1.7%), 11 participants only received one dose of the vaccine (0.9%), 152 participants received two doses of the vaccine (12.3%), 1028 participants received three doses of the vaccine (83.0%), and 27 participants received four doses of the vaccine (2.1%).

Table 3 presents the unadjusted and adjusted models for the associations between PA levels in the last seven days before infections and clinical symptoms. No association was found in the unadjusted model. After adjustment, compared to the low-PA-level group, the moderate-PA-level group presented a higher risk of headaches (OR = 1.34, 95% CI = 1.03 to 1.75, and *p* = 0.03). Compared to the low-PA-level group, the high-PA-level group presented a higher risk of muscle/body aches (OR = 1.42, 95% CI = 1.04 to 1.93, and *p* = 0.03). However, PA levels were not associated with other clinical outcomes (all at *p* > 0.05).

The EQ-5D-5L outcomes are shown in Table 4. The majority of participants reported that they had slight problems with MO (*n* = 400, 32.3%), no problems with SC (*n* = 625, 50.4%), slight problems with UA (*n* = 328, 26.5%), moderate problems with PD (*n* = 388, 31.3%), and no problems with AD (*n* = 438, 35.3%). A small number of subjects reported that they experienced extreme problems with MO (*n* = 72, 5.8%), SC (*n* = 34, 2.7%), UA (*n* = 159, 12.8%), and AD (*n* = 73, 5.9%). Also, only a fraction of individuals had no problems with PD (*n* = 116, 9.3%).

The associations between PA levels in the last seven days before infections and the QoL index value are shown in Table 5. No associations between PA levels and the QoL index value were found on the worst symptomatic day following adjustment for confounders (moderate-PA-level group: β = −0.04, *p* = 0.08; high-PA-level group: β = −0.04, *p* = 0.17).

Table 6 presents the results of multinomial logistic regression for the relationships between PA levels in the last seven days before infections and the five dimensions of EQ-ED-5L. For the MO dimension, the odds (95% CI = 0.28 to 0.95, *p* = 0.03) of having no problems in the moderate-PA-level group was 0.52 times those in the low-PA-level group. Similarly, the odds (95% CI = 0.24 to 0.90, *p* = 0.02) of having severe problems in the moderate-PA-level group was 0.46 times those in the low-PA-level group. In addition, the odds of having no problems (95% CI = 0.24 to 0.93, *p* = 0.03), slight problems (95% CI = 0.24 to 0.93, *p* = 0.03), and severe problems (95% CI = 0.16 to 0.77, *p* < 0.01) in the high-PA-level group were 0.47 times, 0.47 times, and 0.35 times those in the low-PA-level group, respectively. For the UA dimension, the odds of having no problems (95% CI = 0.29 to 0.73, *p* < 0.01) and severe problems (95% CI = 0.36 to 0.96, *p* = 0.04) in the moderate-PA-level group were 0.46 times and 0.59 times those in the low-PA-level group, respectively. Furthermore, the odds (95% CI = 0.33 to 0.98, *p* = 0.04) of having no problems in the high-PA-level group was 0.57 times those in the low-PA-level group.

## 4. Discussion

This study aimed to investigate the associations of PA in the last seven days before COVID-19 infections, clinical symptoms, and the QoL on the worst symptomatic day among individuals infected with mild COVID-19. Our main findings were that individuals with a higher PA level had a greater risk of suffering from headaches and muscle/body aches during mild COVID-19 infections. However, there was no association between PA levels and other clinical symptoms of COVID-19 such as sore throat, fever/chills, or self-perceived fatigue. In terms of the MO and UA dimensions of the EQ-5D-5L, the lower-PA-level group had a lower burden of living on the worst symptomatic day than the higher-PA-level groups did.

Our results showed that PA levels in the last seven days before COVID-19 infections were positively correlated with the odds of developing infectious symptoms (i.e., headaches and muscle/body aches), contrary to our initial hypothesis. Considering that we investigated the PA in the last seven days prior to the COVID-19 infections, the temporary suppression of immune functions may have occurred after engaging in PA, and the continuation of this suppression usually depends on the duration and intensity of PA [24]. Hence, the ineffectiveness of the higher PA levels in relieving infectious symptoms may be explained by the “open window” theory. Some researchers have contended that strenuous exercise bouts or intensified training can impair cellular and humoral immunity, resulting in an increased risk of infections. For instance, relevant studies have reported that salivary IgA which is used to assess whether or not exercise impairs humoral immunity declines in the hours and days after exercise [25]. In addition to decreased salivary IgA, prolonged bouts of high-intensity exercise may also decrease the circulating numbers and functional capacities of leukocytes, thereby lowering immunity [24]. Similarly, Nieman has modeled the correlation between exercise volume and susceptibility to infection in the form of a “J” curve [26]. This model suggests that although moderate exercise can enhance immune function over sedentary levels, excessive prolonged and intense exercise may be immunosuppressive for athletes or adults under high physical stress [27,28,29]. Therefore, the “J” curve model might partly explain the reason why individuals in the higher-PA-level groups have a higher risk of experiencing several symptoms.

The results of this study manifested that the higher PA level prior to COVID-19 infections was associated with an increased risk of experiencing headaches and muscle/body aches. Specifically, compared to the low-PA-level group, the moderate-PA-level group presented a higher risk of headaches and the high-PA-level group presented a higher risk of muscle/body aches. These findings were not consistent with those of several existing studies, which supported that physical inactivity was regarded as a predisposing factor to acquired infections [12,17,30]. For instance, a cross-sectional study assessed the links between PA before COVID-19 infections, disease severity, and symptoms [30] and found that a higher PA level before COVID-19 infection is associated with a reduced risk of moderate illness severity and a reduced risk of suffering from fatigue, dry cough, and chest pain. This may imply that engaging in PA is a helpful way to weaken the severity of COVID-19. One possible reason for these inconsistent results between existing studies and our study may be the differences in the measured PA durations. In their studies, the PA was evaluated as a lifestyle risk factor, which was required to last at least one month or longer, while our study only assessed PA in the last seven days before COVID-19 infections using the IPAQ-SF. Therefore, the difference between habitual behaviors over a month or longer and the recent status of PA may have led to the incongruent results.

This study also found that, compared to individuals in the low-PA-level group, those who were divided into the higher-PA-level groups had an increased risk of obtaining MO and UA problems during the COVID-19 infectious phase. According to the “open window” theory, decreased immunity occurs after conducting strenuous exercise, which may result in a higher risk of developing various discomforting symptoms (e.g., headaches and muscle/body aches) and an easier chance for the invasion of the virus. Consequently, the daily activities of the individuals in the higher-PA-level groups may be limited due to pain to the extent that they have more difficulties in the MO and UA than those with a lower PA level. In addition, the “PA paradox” which describes the potential contrasting health effects of leisure time PA and occupational PA deserves comment [31]. A study recruiting 104,064 individuals from Copenhagen City concluded that higher leisure time PA (e.g., sports, recreation, and transportation) was related to a lower risk of major adverse cardiovascular events and all-cause mortality, while higher occupational PA was correlated with an increased risk [32]. Furthermore, job-related PA accounts for the largest proportion of PA for the majority of the adult population worldwide [33,34]. Although adults occupied a large fraction of the investigated individuals in this study, we applied the IPAQ-SF to measure PA levels, which did not distinguish specific PA domains. As a result, we could not determine whether or not occupational PA and leisure time PA had the opposite effects on participants’ QoL during the COVID-19 infection period. Further studies are needed that use the IPAQ—Long Form (IPAQ-LF) to identify the relationships between different domains of PA and COVID-19-related outcomes.

The main strength of this study is the exploration of the influence of the PA levels before infections on the clinical symptoms and the QoL of mildly infected individuals with COVID-19. However, most previous studies mainly focused on the impact of COVID-19 lockdowns on PA levels among various populations. Nevertheless, several limitations still exist in this study. First, the time point of developing COVID-19 is unpredictable; hence, it is difficult to design a prospective study to explore the influence of PA on COVID-19 infectious symptoms. Therefore, a retrospective self-assessment questionnaire was applied for the PA measurements rather than an objective accelerometer such as ActiGraph, which might have resulted in recall bias and overreporting. Second, in order to improve the completeness of the questionnaire filled out by the respondents, we used the IPAQ-SF rather than the IPAQ-LF to measure PA in the last seven days before infections and participants’ sporting history. However, the “PA paradox” induced by different domains of PA could not be detected with the IPAQ-SF, which limits further discussion about the impact of PA levels on the infectious symptoms and the QoL during the COVID-19 infection period. Third, the results should be interpreted with caution as this is an observational study, which hampers causative relationships. Fourth, details regarding participants’ numbers of infections and variants of infections were not obtained which might have prevented a deeper interpretation of the results. Fifth, using “WeChat” for participant recruitment may have introduced selection bias. Although “WeChat” is widely used in China among a range of users including teenagers to seniors, it is inevitable that subjects were predominantly young individuals due to the complexity of Internet usage.

It is worth mentioning that regular physical exercise has a positive impact on immunity and QoL [35]. For instance, regular exercise can improve the pathogenic activity of tissue macrophages while enhancing the recirculation of immunoglobulins, neutrophils, natural killer cells, cytotoxic T-cells, and immature B-cells. Moreover, PA can also improve immune defense activity and lead to a healthy metabolism [35]. In addition, according to the new 2018 US physical activity guidelines, moving more and sitting less will benefit nearly everyone, and PA of any intensity has health-related benefits although its doses are below the recommended levels [36]. Therefore, the findings in this study do not imply that PA has no protective effects on health-related outcomes. To understand the effect of long-term PA on the QoL and clinical symptoms in mild-COVID-19 patients, the PA levels over a month or even longer need to be investigated in further studies.

## 5. Conclusions

This study suggests that, among mildly infected patients with COVID-19, a higher PA level is associated with higher risks of experiencing several clinical symptoms and a lower QoL. To be specific, higher levels of PA in the last seven days before infections induce a higher risk of experiencing headaches and muscle/body aches. Compared to the lower-PA-level group, the higher-PA-level group has a greater burden of the QoL on the worst symptomatic day.

## Figures and Tables

**Table 1:** Categorization of physical activity levels.

Category	Criteria
1. Low	Individuals who did not meet the criteria for Categories 2 or 3 are considered to have a “low” physical activity level.
2. Moderate	(a) Three or more days of vigorous-intensity activity of at least 20 min per day, or
	(b) Five or more days of moderate-intensity activity and/or walking of at least 30 min per day, or
	(c) Five or more days of any combination of walking, moderate-intensity, or vigorous-intensity activities achieving a minimum total physical activity ≥600 MET-minutes/week.
3. High	(a) Vigorous-intensity activity on at least three days achieving ≥1500 MET-minutes/week, or
	(b) Seven or more days of any combination of walking, moderate-intensity, or vigorous-intensity activities achieving ≥3000 MET-minutes/week.

**Table 2:** Characteristics of the participants.

Variables	
**Age (years), Mean ± SD**	26.02 ± 10.27
<18 years, n (%)	52 (4.2)
18–60 years, n (%)	1177 (95.0)
>60 years, n (%)	10 (0.8)
**BMI (kg/m^2^), Mean ± SD**	22.39 ± 5.35
**Gender, n (%)**	
Female	756 (61.0)
Male	483 (39.0)
**Educational levels, n (%)**	
Primary or below	6 (0.5)
Junior high	41 (3.3)
Senior high	92 (7.4)
College	960 (77.5)
Post-graduate or above	140 (11.3)
**Marital status, n (%)**	
Single	959 (77.4)
Married	265 (21.4)
Divorced	13 (1.0)
Widowed	2 (0.2)
**Employment, n (%)**	
Employed	344 (27.8)
Student	829 (66.9)
Retired	22 (1.8)
Others	44 (3.5)
**History of chronic diseases, n (%)**	
No chronic diseases	1075 (86.8)
At least one chronic disease	164 (13.2)
**Clinical symptoms occurred during the infectious period, n (%)**	
<Seven symptoms	789 (63.7)
≥Seven symptoms	450 (36.3)
**Main exercise places during the COVID-19 pandemic, n (%)**	
Outdoors	236 (19.0)
Indoors	1003 (81.0)
**Vaccination status** **, n (%)**	
Unvaccinated	21 (1.7)
One dose of the vaccine	11 (0.9)
Two doses of the vaccine	152 (12.3)
Three doses of the vaccine	1028 (83.0)
Four doses of the vaccine	27 (2.1)

**Table 3:** Associations between PA levels in the last seven days before infections and clinical symptoms.

Outcome	Exposure	Events (n)	Unadjusted Model				Adjusted Model			
			OR	OR 95% CI		*p* Value	OR	OR 95% CI		*p* Value
Clinical symptom	PA level groups			Lower	Upper			Lower	Upper	
Sore throat	Low (reference)	345	1.00	-	-	-	1.00	-	-	-
	Moderate	283	1.04	0.80	1.35	0.79	1.14	0.87	1.50	0.33
	High	156	0.96	0.70	1.30	0.78	1.12	0.81	1.55	0.49
Fever/chills	Low (reference)	424	1.00	-	-	-	1.00	-	-	-
	Moderate	348	1.07	0.79	1.44	0.68	1.17	0.86	1.60	0.33
	High	199	1.10	0.76	1.59	0.61	1.30	0.89	1.90	0.18
Cough	Low (reference)	419	1.00	-	-	-	1.00	-	-	-
	Moderate	323	0.82	0.62	1.10	0.19	0.91	0.67	1.23	0.53
	High	177	0.73	0.52	1.02	0.06	0.93	0.65	1.32	0.67
Headaches	Low (reference)	322	1.00	-	-	-	1.00	-	-	-
	Moderate	283	1.24	0.96	1.60	0.11	1.34	1.03	1.75	0.03 *
	High	143	0.92	0.68	1.25	0.59	1.04	0.76	1.42	0.83
Self-perceived fatigue	Low (reference)	309	1.00	-	-	-	1.00	-	-	-
	Moderate	266	1.16	0.90	1.50	0.26	1.23	0.95	1.60	0.12
	High	148	1.10	0.81	1.49	0.53	1.29	0.94	1.77	0.12
Anorexia	Low (reference)	221	1.00	-	-	-	1.00	-	-	-
	Moderate	197	1.18	0.92	1.52	0.20	1.23	0.95	1.60	0.11
	High	93	0.87	0.64	1.18	0.36	0.95	0.69	1.31	0.76
Congestion/runny nose	Low (reference)	313	1.00	-	-	-	1.00	-	-	-
	Moderate	265	1.12	0.86	1.44	0.40	1.24	0.95	1.62	0.11
	High	134	0.85	0.63	1.15	0.30	1.00	0.73	1.37	0.99
Muscle/body aches	Low (reference)	241	1.00	-	-	-	1.00	-	-	-
	Moderate	200	1.05	0.81	1.35	0.73	1.08	0.84	1.40	0.55
	High	127	1.30	0.96	1.75	0.09	1.42	1.04	1.93	0.03 *
Loss of smell	Low (reference)	134	1.00	-	-	-	1.00	-	-	-
	Moderate	105	0.96	0.72	1.28	0.77	0.98	0.72	1.32	0.87
	High	55	0.86	0.60	1.23	0.42	0.93	0.64	1.34	0.69
Loss of taste	Low (reference)	151	1.00	-	-	-	1.00	-	-	-
	Moderate	115	0.92	0.69	1.22	0.56	0.94	0.70	1.26	0.68
	High	62	0.86	0.61	1.21	0.38	0.94	0.66	1.35	0.75
Nausea	Low (reference)	94	1.00	-	-	-	1.00	-	-	-
	Moderate	92	1.26	0.92	1.74	0.15	1.39	0.99	1.93	0.05
	High	35	0.78	0.51	1.19	0.25	0.91	0.59	1.40	0.66
Diarrhea/vomiting	Low (reference)	87	1.00	-	-	-	1.00	-	-	-
	Moderate	68	0.96	0.68	1.36	0.81	0.96	0.68	1.37	0.82
	High	43	1.09	0.73	1.63	0.67	1.12	0.74	1.70	0.58
Conjunctivitis	Low (reference)	15	1.00	-	-	-	1.00	-	-	-
	Moderate	5	0.41	0.15	1.12	0.08	0.42	0.15	1.18	0.10
	High	9	1.32	0.57	3.05	0.52	1.47	0.62	3.52	0.39

* *p* < 0.05. Abbreviations: PA (physical activity); OR (odds ratio); CI (confidence interval).

**Table 4:** The QoL on the worst-symptomatic day during the COVID-19 infectious period.

Dimension	Sample
**MO, n (%)**	
No problems	387 (31.2)
Slight problems	400 (32.3)
Moderate problems	238 (19.2)
Severe problems	142 (11.5)
Extreme problems (unable to walk about)	72 (5.8)
**SC, n (%)**	
No problems	625 (50.4)
Slight problems	303 (24.5)
Moderate problems	189 (15.3)
Severe problems	88 (7.1)
Extreme problems (unable to walk about)	34 (2.7)
**UA, n (%)**	
No problems	277 (22.4)
Slight problems	328 (26.5)
Moderate problems	295 (23.8)
Severe problems	180 (14.5)
Extreme problems (unable to walk about)	159 (12.8)
**PD, n (%)**	
No problems	116 (9.3)
Slight problems	271 (21.9)
Moderate problems	388 (31.3)
Severe problems	308 (24.9)
Extreme problems (unable to walk about)	156 (12.6)
**AD, n (%)**	
No problems	438 (35.3)
Slight problems	369 (29.8)
Moderate problems	239 (19.3)
Severe problems	120 (9.7)
Extreme problems (unable to walk about)	73 (5.9)
**EQ-5D-5L index, Mean ± SD**	0.52 ± 0.35
**EQ-5D-5L VAS, Mean ± SD**	48.52 ± 24.29

Abbreviations: MO (mobility); SC (self-care); UA (usual activities); PD (pain and discomfort); AD (anxiety and depression).

**Table 5:** Adjusted linear regression models between physical activity levels in the last seven days before infections and the index value of the QoL.

Outcome	Exposure	β	SE	*p* Value
QoL index value	PA level groups			
	Low (reference)	0.00	-	-
	Moderate	−0.04	0.02	0.08
	High	−0.04	0.03	0.17

Abbreviations: PA (physical activity); SE (standard error); QoL (quality of life).

**Table 6:** Adjusted multinomial logistic regression model between physical activity levels in the last seven days before infections and the dimensions of EQ-ED-5L.

EQ-ED-5L Dimensions	Exposure	β	OR	OR 95% CI		*p* Value
MO †	PA Level Groups			Lower	Upper	
No problems	Low (reference)	0.00	1.00	-	-	-
	Moderate	−0.66	0.52	0.28	0.95	0.03 *
	High	−0.75	0.47	0.24	0.93	0.03 *
Slight problems	Low (reference)	0.00	1.00	-	-	-
	Moderate	−0.49	0.61	0.34	1.12	0.11
	High	−0.75	0.47	0.24	0.93	0.03 *
Moderate problems	Low (reference)	0.00	1.00	-	-	-
	Moderate	−0.31	0.73	0.39	1.37	0.33
	High	−0.72	0.49	0.24	1.00	0.05
Severe problems	Low (reference)	0.00	1.00	-	-	-
	Moderate	−0.78	0.46	0.24	0.90	0.02 *
	High	−1.05	0.35	0.16	0.77	<0.01 **
**SC** †						
No problems	Low (reference)	0.00	1.00	-	-	-
	Moderate	0.10	1.10	0.46	2.62	0.83
	High	−0.62	0.54	0.23	1.26	0.15
Slight problems	Low (reference)	0.00	1.00	-	-	-
	Moderate	0.33	1.39	0.58	3.36	0.47
	High	−0.76	0.47	0.19	1.13	0.09
Moderate problems	Low (reference)	0.00	1.00	-	-	-
	Moderate	0.51	1.67	0.68	4.13	0.27
	High	−0.56	0.57	0.23	1.44	0.24
Severe problems	Low (reference)	0.00	1.00	-	-	-
	Moderate	−0.10	0.91	0.34	2.40	0.84
	High	−0.89	0.41	0.15	1.13	0.09
**UA** †						
No problems	Low (reference)	0.00	1.00	-	-	-
	Moderate	−0.78	0.46	0.29	0.73	<0.01 **
	High	−0.57	0.57	0.33	0.98	0.04 *
Slight problems	Low (reference)	0.00	1.00	-	-	-
	Moderate	−0.30	0.74	0.48	1.14	0.17
	High	−0.38	0.68	0.40	1.16	0.16
Moderate problems	Low (reference)	0.00	1.00	-	-	-
	Moderate	−0.24	0.78	0.50	1.22	0.28
	High	−0.35	0.71	0.41	1.22	0.21
Severe problems	Low (reference)	0.00	1.00	-	-	-
	Moderate	−0.53	0.59	0.36	0.96	0.04 *
	High	−0.46	0.63	0.35	1.15	0.13
**PD** †						
No problems	Low (reference)	0.00	1.00	-	-	-
	Moderate	−0.52	0.60	0.34	1.06	0.08
	High	−0.60	0.55	0.28	1.10	0.09
Slight problems	Low (reference)	0.00	1.00	-	-	-
	Moderate	−0.35	0.70	0.45	1.11	0.13
	High	−0.18	0.84	0.48	1.47	0.54
Moderate problems	Low (reference)	0.00	1.00	-	-	-
	Moderate	−0.08	0.93	0.61	1.41	0.72
	High	−0.32	0.73	0.42	1.24	0.24
Severe problems	Low (reference)	0.00	1.00	-	-	-
	Moderate	−0.31	0.74	0.47	1.15	0.18
	High	0.04	1.04	0.61	1.78	0.89
**DA** †						
No problems	Low (reference)	0.00	1.00	-	-	-
	Moderate	−0.15	0.86	0.48	1.54	0.61
	High	−0.20	0.82	0.43	1.58	0.55
Slight problems	Low (reference)	0.00	1.00	-	-	-
	Moderate	−0.01	0.99	0.55	1.78	0.98
	High	−0.51	0.60	0.31	1.18	0.14
Moderate problems	Low (reference)	0.00	1.00	-	-	-
	Moderate	0.33	1.40	0.76	2.56	0.28
	High	−0.36	0.70	0.34	1.43	0.32
Severe problems	Low (reference)	0.00	1.00	-	-	-
	Moderate	0.37	1.45	0.73	2.85	0.29
	High	0.21	1.23	0.56	2.68	0.60

* *p* < 0.05, ** *p* < 0.01. † Reference: extreme problems (unable to walk about). Abbreviations: PA (physical activity); MO (mobility); SC (self-care); UA (usual activities); PD (pain and discomfort); AD (anxiety and depression); OR (odds ratio); CI (confidence interval).

## Data Availability

The data presented in this study are available on request from the corresponding author. The data are not publicly available due to privacy.
